# Self-care behavior when suffering from the common cold and health-related quality of life in individuals attending an annual checkup in Japan: a cross-sectional study

**DOI:** 10.1186/s12875-015-0300-3

**Published:** 2015-07-29

**Authors:** Fumio Shaku, Madoka Tsutsumi, Asako Miyazawa, Hiroshi Takagi, Tetsuhiro Maeno

**Affiliations:** Department of Internal Medicine, Division of Respiratory Medicine, Nihon University, 30-1 Oyaguchikamimachi, Itabashiku, Tokyo 173-8610 Japan; Faculty of Medicine, University of Tsukuba, Tsukuba, Japan; Graduate School of Comprehensive Human Sciences, University of Tsukuba, Tsukuba, Japan; Yamato clinic, Sakuragawa, Japan

**Keywords:** Self-care behavior, Health-related quality of life, Common cold, Short Form-8™

## Abstract

**Background:**

The World Health Organization and several governments encourage medical self-care (including self-medication) for minor illnesses. Accordingly, the factors that influence self-care have received research attention, with socioeconomic status identified as one such predictor. Although studies have examined the relationship between socioeconomic status and quality of life (QOL) in patients suffering from respiratory allergies or chronic illnesses, the relationship between QOL and self-care behavior for the common cold, the most common illness seen in primary care, has not been examined. Therefore, we investigated the relationship between QOL and self-care behavior in individuals suffering from the common cold.

**Methods:**

We distributed questionnaires to 499 people who attended an annual public health checkup in Kasama city, Japan. Valid questionnaires were received from 398 participants (mean age = 59.0, SD = 15.8, range = 24–87 years; 61.4 % women). The materials included a question relating to typical actions taken when treating a common cold (self-care or visiting a health clinic), demographics, and the Short Form-8™ (SF-8™)—an 8-item survey that assesses health-related quality of life (HRQOL). The association of care action and HRQOL were investigated using Mann–Whitney U tests with a significance level of p < 0.05.

**Results:**

The mean scores for the Physical Functioning, Role-Physical, Bodily Pain, Social Functioning, Role-Emotional, and Physical Component Summary score of the SF-8™ were significantly higher among the self-care group than the group that preferred visiting a clinic.

**Conclusions:**

HRQOL among individuals who engage in self-care when treating the common cold was observed to be significantly higher than among individuals who preferred to attend a health clinic. It is unclear whether self-care behavior affects QOL, or whether QOL affects self-care behavior; however, this finding highlights the importance of the relationship between QOL and self-care behavior. Additional studies should be conducted in order to investigate the direction of causality between self-care behaviors and QOL further.

## Background

Medical self-care may be defined as the range of behaviors undertaken to treat a medical problem without professional intervention [1], and typically represents a preference for use of over-the-counter (OTC) drugs as opposed to clinical visitations. The World Health Organization (WHO) has pointed out that responsible self-medication may help prevent and treat ailments that do not require professional medical consultation [2, 3]. Self-care is encouraged not only as a means to reduce medical expenses, but also for better daily health management and to prevent disease (secondary infection) [4].

Encouragement of self-care, also referred to as patient empowerment, includes giving patients the opportunity to take responsibility and build confidence in their ability to manage their own health [5]. This is typically achieved by facilitating patients’ acquisition of the experience necessary to identifying health needs, setting goals related to personal health care, identifying personal and social obstacles, developing solutions, considering possible consequences of alternative solutions, and making appropriate decisions [6–8]. The empowerment approach is linked to self-determination theory, which supports empowering individuals’ independence in self-care in order to increase the likelihood of formation of enduring self-care habits [9].

Some governments are increasingly encouraging self-care for the treatment of minor illnesses, including self-medication [10]. Recently, the Japanese government has moved in this direction, in an effort to manage rising medical expenses [11]. Therefore, it is now accepted that self-care in the form of responsible self-medication can be beneficial for patients, healthcare providers, the pharmaceutical industry, and governments [4]. Many studies have investigated behaviors affecting self-care, and have identified a number of factors associated with its increased prevalence. The most frequent users of self-care, reported by Figueiras et al., are women, people who live alone, those living in municipalities with over 2,000 inhabitants, and those who left formal education when aged 16 years. For individuals reporting acute disorders, the prevalence of self-medication is greater among those with higher levels of education, while individuals over 43 years of age, those living alone, and students have the highest prevalence of self-medication. Retired people and individuals over 60 years old have the lowest prevalence of self-medication (11.0 % and 11.7 % respectively) [12].

This reported higher prevalence of self-medication in women is in agreement with data reported throughout the literature [13]. Nunes de Melo et al. showed that women and individuals with higher education are more likely to report having used non-prescription medications [14]. Gordon et al. reported that this tendency may be linked, among other factors, to cultural considerations that prompt women to care for themselves and to maintain greater knowledge of pharmaceutical products through more frequent visits to doctors and pharmacists [15]. Nunes de Melo et al. also identified a strong association between poor self-reported health status and non-prescription medication use [14].

A higher prevalence of self-medication has been observed among those living alone [16]; however, this finding differs from those of other studies that have reported that the degree of self-medication correlates with the number of medicines stored at home as a consequence of prescriptions given to other family members [17]. Socioeconomic influences have also been shown to be important and have received attention in the literature. Tobi et al. found that higher socioeconomic status was associated with increased self-care among Dutch adolescents, and that girls reported higher consumption of OTC and prescription drugs (oral contraceptives were excluded) than boys [18]. Low socioeconomic status is generally associated with high psychiatric morbidity, depression [19], and mortality [20]. Furthermore, Turner has found that the risk factors for depression associated with less-effective coping styles, ongoing life events, exposure to stress, and low social support are more prevalent in lower socioeconomic status groups [21].

While many studies have focused on the relationship between self-care and socioeconomic status, others have reported an association between socioeconomic status and quality of life (QOL) among patients suffering from respiratory allergies [22] and osteoporosis [23], for example. In addition, QOL and psychopathological problems are strongly negatively associated [24, 25], while psychological distress has been identified as an important contributor to QOL. This suggests that methods of reducing psychological distress, such as improving social support and sleep, are also likely to positively impact QOL [26]. In support of this notion, a number of studies have demonstrated an association between QOL and physical illness [27], indicating that greater medical burden is associated with increased risk of depression [28, 29].

Despite the well-documented relationship between QOL and socioeconomic status, no study to date has directly examined associations between QOL and self-care behavior. A Japanese article [30] indirectly explored the relationship between self-care and QOL among diabetic patients . The authors measured self-care behavior, self-esteem, and subjective well-being, and reported that self-care behavior is related to increased subjective well-being and self-esteem. By extension, it was reasoned that subjective well-being and self-esteem are related to QOL, and the authors therefore concluded that positive self-care behavior is closely associated with higher QOL.

The purpose of this study was therefore to address this gap in the literature to promote more widespread use of self-care. We hypothesized that QOL would be related to self-care behavior in treatment of the common cold. This viral disease was selected as the focus of the study, as previous studies have reported that throat-related symptoms and bronchitis were the most common reasons for self-medication [31–33], and because participants could reasonably be assumed to be familiar with the common cold.

## Methods

The Human Subjects Institutional Review Board of the University of Tsukuba (No. 681) approved this study. The purpose and contents of the questionnaires were individually explained to potential participants by the researchers before written informed consent was received.

### Participants

This cross-sectional study was conducted during a period of one month at the largest checkup center in Kasama City, Japan, which has a population of approximately 77,000 people. In Japan, annual medical checkups (which include blood and urine tests, blood pressure checks, EKG, and chest X-rays) are free of charge and, while not compulsory, Japanese are requested to attend checkups yearly. Prospective participants were excluded if they were unable to complete the survey due to physical or cognitive impairment.

A total of 499 questionnaires were distributed, with 451 being returned along with informed consent. Of this number, 62 questionnaires were incomplete and were excluded, leaving a final sample of 389. All participants were native Japanese.

### Instruments

The set of questionnaires (Table 1) contained questions examining demographic characteristics and common cold treatment behaviors, and the Japanese version of the SF-8™.

**Table 1:** Questionnaire (translated into English)

How old are you?
Are you male, or female?
How many family members do you live with?
Do you visit a clinic regularly?
Do you take medicine regularly?
Do you keep medicine for the common cold in your home?
If you catch a cold (having symptoms but not interfering with daily life), which do you prefer?
immediately visit a clinic
maybe immediately visit a clinic
first attempt self-care
maybe first attempt self-care
SF-8
8 questions (license needed)

Common cold self-care behaviors were assessed with the question, “Do you (maybe) immediately go to a clinic, or (maybe) first attempt self-care upon catching the common cold?” (see Table 1).

The SF-8™ is an 8-item survey that assesses health-related QOL (HRQOL), which has been translated into Japanese and validated by Fukuhara et al. [31–33]. The instrument is widely used in the evaluation of allopathic treatment modalities and has been used in many Japanese studies. We obtained permission to use the questionnaire from the Medical Outcomes Trust, Health Assessment Lab, Quality Metric Incorporated, and Shunichi Fukuhara. The tool measures eight categories: Physical Functioning (PF), Role-Physical (RP), Bodily Pain (BP), General Health (GH), Vitality (VT), Social Functioning (SF), Role-Emotional (RE), and Mental Health (MH). The first four categories assess physical aspects of HRQOL and result in a Physical Component Summary (PCS) score; the latter four assess mental and psychosocial aspects of HRQOL and result in a Mental Component Summary (MCS) score. Scoring is based on Japanese standards (Japanese norm-based scoring compared with standardized Japanese scores); possible subscale scores range from 0 to 100, with higher scores indicating a better QOL [31]. We calculated Japanese norm-based subscale, PCS, and MCS scores using an SF-8™ scoring program (iHope International corporation, Kyoto, Japan) provided by the Institute for Health Outcome and Process Evaluation Research [31].

### Data collection

Questionnaires were distributed to all individuals who attended the check-up center during the data collection period. Participants were asked to complete the questionnaires within the check-up center and return them in an envelope, along with their written informed consent, before leaving.

### Data analysis

Participants were divided into two groups: Group I (prefer to first attempt self-care, prefer to maybe first attempt self-care) and Group II (prefer to immediately visit a clinic, prefer to maybe immediately visit a clinic).

Review of SF-8™ subscales and component scores indicated that none was normally distributed. We therefore analyzed the data using Mann–Whitney U tests, with a significance level of p < 0.05. Additionally, multiple logistic regression analysis was conducted using the forward selection method in order to investigate potential interactions between factors.

All analyses were conducted using SPSS ver. 21 (IBM, Tokyo, Japan).

## Results

### Demographic variables

Participants’ mean age was 59.0 years (SD = 15.8), with a range of 24–87 years. Complete questionnaires were received from 150 men (38.6 %) and 239 women (61.4 %), and approximately two thirds of all participants (63.5 %) classified themselves as preferring self-care. Of these, 252 (64.8 %) had the medicines stored at their residence and, 18 (4.7 %) had three or more medicines stored.

### Outcomes

The mean age and ratio of participants in both groups are shown in Table 2. The results of a *t*-test revealed that the mean age of Group II was significantly higher than that of Group I (p < 0.001), indicating that younger persons are more likely to report that they would engage in self-care when suffering from a common cold rather than consult a doctor. Results of a chi-squared test also showed that the percentage of males in Group II was higher than in Group I.

**Table 2:** SF-8 scores by group

		Group I	Group II	p value	
		(n = 247)	(n = 142)		
Age		56.5 ± 15.7	63.4 ± 15.2	<0.001*	*1
Male		89 (36.0 %)	61 (43.0 %)	0.195	*2
SF-8 norm-based mean score	PF	49.9 ± 6.7	48.2 ± 7.2	0.001*	
	RP	50.0 ± 6.4	48.5 ± 6.9	0.008*	
	BP	52.8 ± 7.8	50.1 ± 8.2	0.001*	
	GH	50.0 ± 6.2	50.2 ± 6.8	0.596	*3
	VT	51.2 ± 6.0	51.1 ± 6.1	0.844	
	SF	50.8 ± 7.4	48.8 ± 8.6	0.016*	
	RE	50.1 ± 5.2	48.3 ± 7.5	0.041*	
	MH	50.6 ± 6.0	49.7 ± 7.3	0.521	
	PCS	49.6 ± 6.7	48.0 ± 6.3	0.006*	*1
	MCS	49.7 ± 6.0	48.8 ± 7.8	0.659	

*: significant difference

*1: *t*-test

*2: chi-squared test

*3: Mann–Whitney *U* test

Group I: Prefer to first attempt self-medication

Group II: Prefer to immediately visit a clinic

Subscales: Mann–Whitney U-test

PCS and MCS: *t*-test

The mean scores and significance values of the eight components of the SF-8™ by group are also shown in Table 2. Mann–Whitney U tests revealed that, with the exception of General Health and Vitality (which were not found to differ significantly by group), the mean scores of all subscales except General Health and its components were higher in Group I than in Group II. The mean scores of Physical Functioning, Role-Physical, Bodily Pain, Social Functioning, Role-Emotional, and Physical Component Summary were significantly different (p < 0.05) across the groups.

In order to investigate possible interactions between experimental factors, we performed multiple logistic regression analysis using the forward selection method. Odds ratios of age, Bodily Pain, and Role-Emotional were 1.033, 0.971, and 0.950, respectively (Table 3).

**Table 3:** Multiple logistic regression analysis of factors related to self-care behavior

Independent variable	Odds ratio	95 % Cl
Age	1.033	1.018–1.048
BP	0.971	0.945–0.999
RE	0.950	0.915–0.985

## Discussion

This is the first study to explore the relationship between self-care behavior when suffering from the common cold and HRQOL. Previous studies have explored associations between self-care and other factors including education, socioeconomic status, and lifestyle. Yokoyama et al. indirectly studied the relationship between self-care and QOL for diabetes patients [30]; however, no study has explored the relationship between self-care and HRQOL directly. In addition to confirming our hypothesis that a relationship exists between HRQOL and self-care behavior for the treatment of the common cold, we aimed to promote awareness of this association in order to increase prevalence of self-care behaviors.

In this study, behaviors for treating the common cold were selected as representative of general self-care behavior, and were compared between two groups (self-care and clinic). Scores on five subscales of the SF-8™ (PF, RP, BP, SF, and RE), and PCS scores, varied significantly between groups, with results indicating that HRQOL as measured by these scales is higher among individuals who practice self-care. The subscales of GH, VT, MH, and MCS did not significantly differ between groups. Interestingly, these subscales primarily relate to psychological factors, or are subscales that tend to be affected by psychological factors. Additionally, in multiple logistic regression analysis, odds ratios of age, BP, and RE were 1.033, 0.971, and 0.950, respectively. This suggests that age and some components of HRQOL are related to self-care behavior.

Younger participants were found to be more likely to engage in self-care. This may be due to greater physical strength among younger people, as well as a tendency for elderly people to be more concerned about contracting serious diseases, such as pneumonia.

Economic variables have been repeatedly shown to influence QOL. Specifically, medical insurance may affect prevalence of and preference for self-care. In Japan, public health insurance is available to everyone; therefore, citizens who visit clinics for treatment of common colds do not pay a premium. Additionally, the cost of prescription drugs is lesser than that of OTC drugs, as a result of Japan’s medical insurance system. This is particularly true among elderly people (65 years and over). It is therefore likely that this health insurance system affects individuals’ self-care behavior; hence, further research is required examining economic factors related to self-care.

It is illuminating to compare the Japanese medical system to systems implemented abroad. The Japanese medical insurance system is characterized by provisions for universal medical care, nursing care for older persons, and payment of health care expenses [34]; large differences thus exist between the US and Japanese culture and medical practices, which may affect self-care behaviors. In the United States, for example, private insurance companies provide most medical insurance, with the outcome that wealthy people are able to obtain very advanced, but expensive, medical care, whereas the uninsured poor can only afford a limited amount of the available medical services [35]. In Germany, by contrast, patients are permitted to freely choose their general practitioner, but are prevented from changing practitioners for at least three months after the first visit, unless there is a special reason for doing so [36]. Additionally, access to German hospital specialists is subject to referral by the patient’s primary physician, which often takes a long time. Patients that consult a specialist without a referral are expected to pay the cost of subsequent medical treatment. Finally, in the United Kingdom, access to physicians and medical institutions is controlled by a registration system [37]. All patients must register in the system through a general practitioner; patients are referred to a hospital only after first being examined by that physician, except in emergencies [34].

While the Japanese system is most similar to that of the UK, significant differences exist. The UK’s system is nationalized, although nationalization is divided between purchasing and delivering care, whereas Japan maintains a social insurance system with 80 % of hospitals remaining private. Nonetheless, Japan’s final healthcare expenditure, regarding cost containment, is similar to that of the UK [38].

Yokoyama et al. reported that subjective well-being and self-esteem are related to QOL, and that positive self-care behavior is closely indirectly associated with high QOL [30]. The findings of this present study extend these results, not only by directly measuring QOL, but also by examining self-care for the common cold. This study’s findings may therefore be more generalizable than those of studies only directly measuring QOL.

Future research should examine economic factors related to self-care, as these have been shown to influence QOL and may strongly affect self-care behavior. Additionally, self-care behaviors for the common cold may be related to the use of stored medication. Sugawara reported [39] that the practice of self-medication is correlated with having three or more medicines stored at home. We asked our participants to report the number of medications stored in their home, and found that 9.97 % kept three or more. We therefore suggest that the number of stored medicines was not a significant factor in our study. Finally, future studies of self-care behavior in the Japanese population should examine variables known to affect self-medication, in order to better explore self-medication as a subset of self-care behavior. Understanding why some individuals may choose to self-care while others visit a clinic may illuminate cost as a potential mediator of self-care behavior. In addition, individual differences such as personality and illness-perception may influence self-care behavior. A multinational comparison of self-care behaviors in Japan with those of other countries would extend understanding of the mechanisms affecting self-care.

### Limitations

We used self-administered questionnaires to collect data; this measure type is inherently limited and open to response bias. Further, data were collected from a single checkup center in one city in Japan; hence, the sample may not have been representative. In general, Japanese workers often receive health examinations at their workplace (private periodic checkups); only individuals without this facility attend public checkups. This may account for the large number of female participants in the sample. Furthermore, checkups are more likely to be attended by those individuals who are more concerned about their health; hence, the sample may have tended to engage in more healthcare practices than the general population. Additionally, previous studies have reported the existence of many other variables connected to self-care behaviors that were not were controlled for in the present study. These limitations mean that our results may not be generalizable to the general population of Japan, or to other countries. In light of these limitations, future studies should address self-care using more diverse and robust measures of healthcare practices, better control for variables that may affect attendance of various types of clinics, and examine more representative samples. Research could also usefully examine whether this study’s findings are replicated outside the common cold.

## Conclusions

HRQOL was found to be higher among individuals who prefer to self-care when treating the common cold than among those who prefer to visit a health clinic. Whether self-care behavior affects HRQOL, or HRQOL affects self-care behavior, cannot be inferred from this study’s results, as a cross-sectional methodology was used. Future research should address this topic with designs that are able to determine causation.

This study was the first to investigate the association between self-care behavior and HRQOL in treating the common cold. In addition, our examination of the HRQOL subscales indicated that physical components of HRQOL are more strongly associated with self-care behavior than mental health components.

## Abbreviations

WHO: World Health Organization
QOL: Quality of life
HRQOL: Health-related quality of life
OTC: Over-the-counter medication
SF-8™: Short Form-8™
PF: Physical Functioning
RP: Role-Physical
BP: Bodily Pain
GH: General Health
VT: Vitality
SF: Social Functioning
RE: Role-Emotional
MH: Mental Health
PCS: Physical Component Summary
MCS: Mental Component Summary
